# Post-COVID Postural Orthostatic Tachycardia Syndrome and Inappropriate Sinus Tachycardia: Prevalence, Overlap, and Clinical Characteristics

**DOI:** 10.1016/j.jacadv.2026.102702

**Published:** 2026-04-02

**Authors:** Maria Juszczyk, Sara Nawaz, Ali Mahdi, Christian Lewinter, Fabrizio Ricci, Marcus Ståhlberg, Artur Fedorowski

**Affiliations:** a Department of Cardiology, Karolinska University Hospital, Stockholm, Sweden; b Department of Medicine, Karolinska Institute, Solna, Stockholm, Sweden; c Department of Neuroscience, Imaging and Clinical Sciences, G. D Annunzio University of Chieti-Pescara, Chieti, Italy; d Department of Clinical Sciences, Malmö, Lund University, Malmö, Sweden

**Keywords:** cardiovascular autonomic dysfunction, inappropriate sinus tachycardia, long COVID, postural orthostatic tachycardia syndrome

**Figure:**
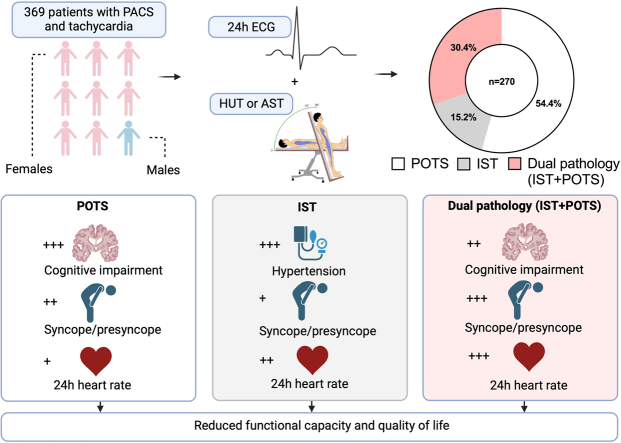

As the world recovered from the COVID-19 pandemic, new and complex patient cases emerged, revealing longstanding and previously unpredictable cardiac manifestations. In this context, cases of postural orthostatic tachycardia syndrome (POTS) and inappropriate sinus tachycardia (IST) have been extensively reported. Although separate diagnostic definitions have been established, the clinical delineation between the disorders remains imprecise.^1^ This study aimed to characterize and compare clinical and hemodynamic profiles of POTS and IST in long COVID patients.

**What is the clinical question being addressed?**

What are the clinical and hemodynamic differences between postural orthostatic tachycardia syndrome and inappropriate sinus tachycardia?

**What is the main finding?**

Inappropriate sinus tachycardia exhibits higher rates of hypertension, whereas postural orthostatic tachycardia syndrome and dual pathology exhibit higher rates of presyncope, cognitive impairment, and nausea.

POTS is defined by a heart rate (HR) increase of at least 30 bpm within 10 minutes of assuming an upright position or by using a tilt-table, absence of orthostatic hypotension, reproduction of symptoms during testing, and a history of at least 3 months of orthostatic intolerance and POTS symptoms.^2^ IST is defined by an average HR above 90 bpm using 24-hour electrocardiogram (ECG) monitoring or as a resting HR exceeding 100 bpm at more than 1 occasion. Both definitions require exclusion of other causes of sinus tachycardia.^3^

## Study population and methods

**Figure 1:**
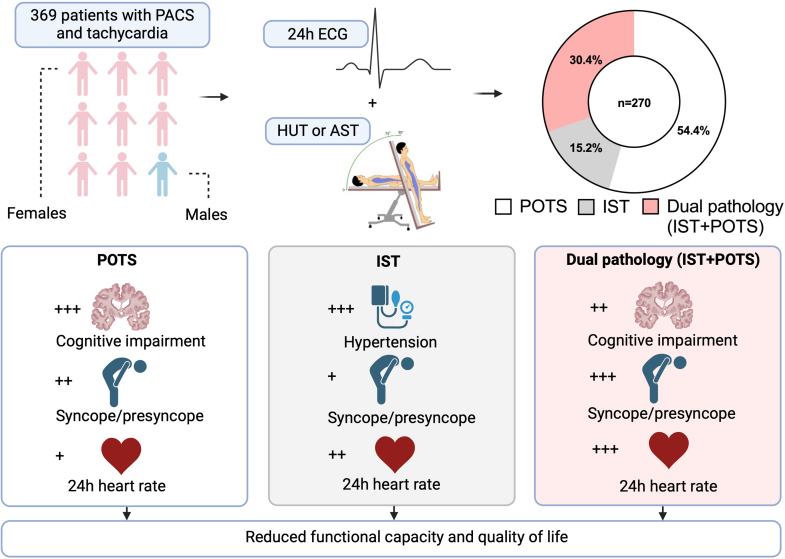
**Study Inclusion and Group Characteristics** Inclusion process and final participant count; proportions of POTS, IST and dual pathology; clinical characteristics by group. AST = active standing test; ECG = electrocardiogram; HUT = head-up tilt; IST = inappropriate sinus tachycardia; PACS = post-acute COVID-19 syndrome; POTS = postural orthostatic tachycardia syndrome.

A cross-sectional study was conducted on patients referred to the Dysautonomia and Syncope Unit, Department of Cardiology, Karolinska University Hospital, Stockholm (2020-2024) due to suspected post-COVID sinus tachycardia. The patients were selected on the combination of unexplained sinus tachycardia and long COVID, defined as an infection-associated chronic condition that occurs after SARS-CoV-2 infection and is present for at least 3 months as a continuous, relapsing and remitting, or progressive disease state that affects 1 or more organ systems (Figure 1).^4^ As seen in Figure 1, study inclusion required a baseline 24-hour Holter ECG and an orthostatic test (active standing test/head-up tilt test). Pharmacological agents with known effects on the cardiovascular system were paused during testing. The study included 270 patients divided into a POTS group, an IST group and a dual pathology group (POTS + IST). The implementation of this study has been approved by the Swedish Ethical Review Authority.

Pharmacological treatment was considered relevant if it was prescribed specifically for treating POTS/IST, if there was no report of treatment discontinuation, and if the prescribed treatment had been deemed effective either by subjective symptom relief or by decreased 24-hour average HR or diminished increase in HR upon standing.

Categorical variables were expressed as frequencies and percentages. Continuous variables were assessed for distributional form; approximately normally distributed variables were reported as mean ± SD, asymmetrically distributed variables were reported as median (IQR). Group comparisons across the 3 phenotypes (POTS, IST, and dual pathology) were performed using the Pearson chi-square test (Fisher exact test for frequencies <5) for categorical variables, one-way analysis of variance for normally distributed continuous variables, and the Kruskal-Wallis test for non-normally distributed continuous variables. Where multiple pairwise comparisons were undertaken following an overall significant group test, *P* values were adjusted using a Bonferroni correction. A 2-sided *P* value <0.05 was considered statistically significant. Statistical analyses were performed using R (version 4.2.0, R Foundation for Statistical Computing) or SPSS (version 28.0; IBM).

## Results

The cohort included 147 POTS patients (mean age 38 ± 10 years), 41 IST patients (mean age 45 ± 12 years), and 82 dual pathology patients (mean age 39 ± 13 years; *P* < 0.0001). Hypertension was more prevalent in IST patients (36.6%) compared to POTS (9.5%) and dual pathology (20.7%; *P* < 0.001). Presyncope/syncope was most frequently reported in dual pathology (POTS 58.5%; IST 46.3%; dual pathology 69.5%; *P* = 0.04). Nausea was more common in POTS (63.3%) than in IST (36.6%; *P* = 0.004). All groups reported similar symptom burden according to the mean Malmö POTS Symptom Score (MAPS; POTS 61 points, IST 61 points, dual pathology 66 points). Palpitations were the most common symptom (POTS 90.5%; IST 85.4%; dual pathology 93.8%).

Resting systolic blood pressure was slightly higher in IST (124 ± 18 mm Hg) compared to POTS (117 ± 14 mm Hg; *P* = 0.001) but comparable to dual pathology (124 ± 12 mm Hg). Functional exercise capacity, assessed by 6-min walk test, was reduced across all groups (POTS 430 ± 162 m; IST 461 ± 162 m; dual pathology 421 ± 130 m; *P* = 0.221; normal range 580-610 m). Left ventricular ejection fraction was preserved and comparable across all groups.

Ivabradine was the most frequently prescribed pharmacological agent, followed by beta-blockers, with comparable use across all groups. Pyridostigmine (POTS 42.2%; IST 12.2%; dual pathology 34.1%; *P* = 0.003), fludrocortisone (POTS 10.9%; IST 0%; dual pathology 3.7%; *P* = 0.02) and midodrine (POTS 24.0%; IST 7.3%; dual pathology 13.4%; *P* = 0.02) were all more frequently prescribed in the POTS group.

## Discussion

The diagnosis of POTS (54.4%) and dual pathology (30.4%) were more common than isolated IST (15.2%). Dual pathology affected 30.4% of the study population, which strengthens the theory of shared pathophysiology of the 2 disorders. All groups appeared equally affected by their symptoms according to MAPS. The assumption of equal impact level was further supported by the similar results observed in the 6-min walk test and exercise ECG, used to assess functional exercise capacity and endurance.^1^^,^^2^ When analyzing the 2 subcategories in the MAPS survey, noncardiac symptoms were reported to be more challenging than cardiac symptoms. However, the most frequently reported symptoms were palpitations and dyspnea, which are considered cardiac symptoms. These contrasting results of frequency and intensity represent the complexity of post-COVID sinus tachycardia symptomatology.

Patients with IST exhibited higher rates of hypertension and higher mean systolic blood pressure at rest in comparison to POTS and dual pathology, suggesting a potential significance of hypertension screening in patients with IST. An elevated HR, as seen in IST patients, is often associated with hypertension and cardiovascular morbidity, and may be an expression of primary hyperadrenergic drive that is not dependent on hypovolemia, corresponding to one of the proposed POTS phenotypes. Elevated HR has also been considered to be a biomarker of cardiac activation of the sympathetic nervous system in patients with essential hypertension.^5^ Reduction in chronotropy was the most frequently targeted treatment mechanism in our study population, reflected by the predominant use of ivabradine and beta-blockers.

Among patients with long COVID and sinus tachycardia, POTS and IST represent distinct but overlapping cardiovascular autonomic phenotypes. However, IST exhibits clinical and hemodynamic profiles with higher rates of hypertension and higher resting systolic blood pressure, whereas POTS and dual pathology exhibit higher rates of presyncope, cognitive impairment, and nausea. These findings highlight the heterogeneity of long COVID cardiac manifestations, underscoring the need for tailored diagnostic and therapeutic approaches. Our study included patients referred to a tertiary center due to suspected post-COVID sinus tachycardia, which may introduce selection bias compared with the general population.

## Funding support and author disclosures

Artur Fedorowski was supported by the Swedish Heart Lung Foundation (Grant no 20220317), and Swedish governmental funding of clinical research (ALF) for Region Stockholm (Grant no 988024). The remaining authors have no relationships relevant to the contents of this paper to disclose.
